# Trajectories of housing affordability and mental health problems: a population-based cohort study

**DOI:** 10.1007/s00127-022-02314-x

**Published:** 2022-06-29

**Authors:** Kate Dotsikas, David Osborn, Kate Walters, Jennifer Dykxhoorn

**Affiliations:** 1 Division of Psychiatry, University College London, London, W1T 7NF UK; 2 Department of Primary Care and Population Health, University College London, London, UK

**Keywords:** Housing affordability, General Health Questionnaire-12, UK, Group-based trajectory modelling, Cohort study

## Abstract

**Purpose:**

With housing costs increasing faster than incomes and a limited supply of social housing options, many households face unaffordable housing. Housing affordability problems may negatively impact mental health; however, longitudinal evidence is limited. This study investigates the association between trajectories of housing affordability problems and mental health.

**Methods:**

We used data from 30,025 households from Understanding Society, a longitudinal household survey from the UK. Participants spending 30% or more of household income on housing were categorised as facing housing affordability problems. We estimated group-based trajectories of housing affordability problems from 9 waves of data (2009–2019). We used linear regression to calculate the association between the trajectories and mental health problems, as measured by General Health Questionnaire (GHQ) score in Wave 10 (2018–2020).

**Results:**

We found six distinct trajectories of housing affordability problems. Those in the ‘stable low’ group had a consistently low probability of affordability problems, whilst those in ‘high falling’ group had a sustained high probability in the earlier waves of the study, subsequently decreasing over time. The adjusted analysis showed that trajectory group membership over the first nine waves of data predicted GHQ score in 2018–2020 (Wave 10). Compared to the ‘stable low’ group, those in the ‘high falling’ group had a GHQ score that was 1.06 (95% CI 0.53–1.58) points higher.

**Conclusion:**

This study provides evidence that sustained exposure to housing affordability problems is associated with long-term worse mental health, even in the absence of more recent problems.

**Supplementary Information:**

The online version contains supplementary material available at 10.1007/s00127-022-02314-x.

## Introduction

Since the 2008 financial crisis, a lack of consistent access to affordable housing has emerged as a global problem. Some of the highest rates of unaffordable housing in Europe can be found in the UK [1], with over 2.5 million people facing affordability problems in England alone [2]. The transformation of housing into a financial investment rather than a basic right to shelter has priced out many from the housing market [3, 4]. Simultaneously, the cost of rent has risen faster than income. Income growth in the UK has slowed in recent years, with median income increasing on average − 0.3% per year from 2016–2017 to 2018–2019 [5], whilst private rental prices have increased by 10.3% since 2015 [6]. Finally, the availability of social housing has been in decline since the introduction of the Right-to-Buy schemes in the 1980s; there are now approximately 1.5 million fewer social homes today compared to 1980 [7]. Between the unattainability of owning a home, rising cost of rent relative to income, and the limited availability of social housing, a growing population has been left with few affordable alternatives, and a large proportion of their household income is consumed by housing costs.

Housing affordability problems may pose a risk to mental health. There is a large body of research linking physical housing conditions and mental health [8–10]. A small number of papers have found that housing affordability problems are associated with poor mental health [11–15], as measured by self-reported general mental health scales, including the Short Form Health Survey mental health component and the General Health Questionnaire (GHQ). However, few studies have conducted longitudinal analyses since the 2007–2009 global financial crisis, nor have they examined the role of dynamic housing affordability pathways on mental health.

At a given point, many people may be exposed to unaffordable housing, but without longitudinal analysis, it is impossible to distinguish between those who are experiencing cumulative unaffordable housing or intermittent problems and their associated mental health impact. Baker et al. found a dose–response effect of housing affordability on mental health in an Australian cohort, with both prolonged and intermittent exposure associated with an increasing negative impact on mental health [15]. This research suggests that different patterns of affordability problems over time may present distinct relationships with mental health outcomes. The existing studies assume exposure patterns, most commonly using three pre-existing categories of cumulative problems, intermittent problems, and no problems. However, the intermittent category could be very diverse, including those who have an increasing risk of housing affordability problems, a decreasing risk, or more unstable trajectories. No study has taken a more nuanced approach and examined the mental health impact of housing affordability trajectories, which allows us to investigate and describe the underlying unique trajectories in the population.

This study aims to determine the longitudinal relationship between housing affordability over time and mental health. Specifically, we will estimate the trajectories of housing affordability problems and examine the relationship between these trajectories and mental health. We expected to find that cumulative housing affordability problems over time would be associated with worse mental health, compared to individuals with little to no affordability problems. Similarly, trajectories of intermittent affordability problems would be associated with worse mental health compared to individuals consistently exposed to few or no affordability problems.

## Methods

### Data

We used data from Understanding Society, the United Kingdom Household Longitudinal Survey (UKHLS). UKHLS is a nationally representative panel survey that collects information annually from individuals and households in England, Scotland, Wales, and Northern Ireland [16]. UKHLS used stratified, clustered equal probability sample design to select the study sample, with approximately 40,000 households responding at baseline in 2009 [17]. Information for our study was taken from 2009–2011 (Wave 1 of UKHLS) until 2018–2020 (Wave 10 of UKHLS). Whilst data collection for each wave occurs over a 2-year period, the interval between questionnaires for each individual participant is 1 year.

### Participants

The analysis is based on a sample of UKHLS participants that responded to the survey at Wave 1 (2009–2011). We selected one individual from each household as the reference person, assuming this person may bear more responsibility for household finances. This person is the owner or renter of the accommodation in which the household lives and is most often the person who answers the household questionnaire. If there were multiple owners or renters, the eldest was selected.

### Measures

Our primary outcome was mental health problems, measured using the 12-item GHQ in 2018–2020 (Wave 10). Participants score between 0 and 4 on each item, resulting in a continuous Likert score between 0 and 36, where 0 indicates no mental health problems and 36 indicates worse mental health. In our study, GHQ was treated as a continuous score. Previous studies have shown that GHQ sores of 12 or higher are consistent with a diagnosis of a common mental disorder [18, 19].

The main exposure was trajectories of housing affordability problems. Housing affordability problems were measured using housing cost burden. The percentage of net equivalised household income spent on housing costs (rent or mortgage) was calculated at each wave, and a binary variable marked by a threshold of 30% of income spent on housing costs was used; individuals spending 30% or more on housing costs were categorised as having a housing cost burden, and therefore facing housing affordability problems. Whilst there is no gold standard of housing cost burden measures, the 30% cut-off is the most widely used, including by the Office for National Statistics [20]. This measure is a reliable indicator of housing stress and is straightforward to use and for policymakers to understand [21]. Covariates from Wave 1 included age, sex, country (England; Scotland; Wales; Northern Ireland), and low income (binary cut-off above or below median household income). Ethnicity (White British/Irish; Other white background; Mixed background; Indian; Pakistani; Bangladeshi: Black Caribbean; Black African; Other non-white background) and GHQ at Wave 1 were not controlled for in the main analysis, but were used to describe the trajectory groups.

### Derivation of group-based trajectories for housing affordability problems

We used group-based trajectory modelling to determine sub-groups following distinct clusters of housing affordability problems trajectories across Waves 1–9 of UKHLS. Using the Stata package TRAJ [22], we fitted a series of weighted models with increasing numbers of trajectory clusters. The data were modelled using logistic regression, with the derived trajectories representing the probability of being categorised as having housing affordability problems across the study waves. The optimal polynomial functions for each number of trajectory clusters were determined using the approach laid out by Andruff et al., wherein higher-order polynomials are removed when there is little evidence to reject the null hypothesis that the parameter estimate is equal to zero [23]. Finally, we fit a series of models with increasing numbers of clusters, comparing the Bayesian information criterion (BIC) to assess model fitness [23]. Participants with at least one data point of housing affordability problems were included in the analysis. The trajectory analysis was repeated using participants with complete housing affordability data as a sensitivity analysis to account for the potential impact of attrition.

We determined the distribution of the sample characteristics within each trajectory group and compared the different values using chi^2^ and *F* test for categorical and continuous variables, respectively.

### Housing affordability problems trajectories and mental health problems

We used linear regression to estimate the relationship between our housing affordability problems trajectories and mental health problems as indicated by GHQ score. The regression was set to account for the primary sampling unit and strata of the multi-stage sampling design. We compared three linear regression models: an unadjusted model (model I), a partially adjusted model adjusting for age, sex, and country at baseline (model II), and a fully adjusted model adjusting for model II covariates plus low income (model III). As mental health is time-varying, including the GHQ score at a single time point to adjust for pre-existing mental health in the linear regression would be arbitrary and not capture how mental health varied, considering the 10-year follow-up period. However, we conducted a sensitivity analysis additionally adjusting for Wave 1 GHQ to represent pre-existing mental health, as well as a sensitivity analysis adjusting for ethnicity.

### Probability weights

To account for the unequal selection probabilities, differential non-response, and potential sampling error in UKHLS, we used probability weights to ensure estimates are representative of the UK population. Of the identified cohort, only 30.9% (*n* = 9284) had full or partial responses to our variables at all waves, rendering the panel unbalanced; cohort members did not necessarily respond at every wave. Therefore, an additional adjustment accounting for the probability of being a respondent at all waves was included.

### Imputation of missing data

The data were assumed to be missing at random. TRAJ uses maximum likelihood estimation to estimate the parameters, allowing all available information to be used from each subject to account for potential bias due to missing data. Missing GHQ and covariate data were imputed using multiple imputations with chained equations in wide format. The imputation was weighted with the same weighting approach as the analysis to ensure compatibility. Any item or unit non-responses on analysis variables were imputed, as well responses of ‘don’t know,’ ‘refusal,’ and ‘proxy.’ All of the variables in the regression model were used in the imputation model, with the addition of ethnicity and GHQ scores from all nine waves included as auxiliary variables. A sensitivity analysis was performed to compare the results of the linear regression using imputed data and complete cases.

All analyses took place in STATA 16.

## Results

### Sample characteristics

The final analysis was based on a sample of 30,025 participants. This sample excluded any proxy respondents at Wave 1; these respondents almost never provided mental health data across all data waves. The final sample also excluded three participants who never provided ethnicity data. The sample exclusions can be found in greater detail in the supplemental material (Supplemental material, F1). The distribution of sample characteristics can be found in Table 1. The total sample contained approximately equal numbers of males and females, with a mean age of approximately 50. The majority of the sample was White British/Irish, from England, and was categorised as not having a housing affordability problem at Wave 1.

**Table 1:** Distribution of sample characteristics by trajectory group

Variable	Total (*n* = 30,025)	Stable low (*n* = 15,791)	Stable moderate (*n* = 1983)	Steady increase (*n* = 1847)	Rapid decrease and slight increase (*n* = 2089)	Stable high (*n* = 6936)	High falling (*n* = 1379)	Chi-square or *F* statistic (*p* value)
GHQ (Wave 1) —mean (SD)	11.3 (5.5)	10.8 (5.1)	11.3 (5.5)	11.6 (5.7)	12.1 (5.9)	11.9 (6.1)	12.02 (6.1)	47.2 (< 0.001)
GHQ (Wave 10)—mean (SD)	11.2 (5.5)	10.4 (4.8)	11.7 (5.9)	11.8 (5.5)	11.4 (5.7)	12.2 (6.3)	12.4 (6.2)	44.4 (< 0.001)
Housing cost burden (Wave 1)—mean (SD)	10,718 (35.7)	633 (4.0)	93 (4.7)	365 (19.8)	2013 (96.4)	6626 (95.5)	988 (71.7)	19,103.6 (< 0.001)
Age—mean (SD)	50.0 (17.0)	57.3 (16.4)	46.4 (14.0)	42.5 (14.4)	43.7 (13.6)	39.8 (13.2)	42.9 (12.3)	1628.2 (< 0.001)
Female—*n* (%)	15,393 (51.3)	7758 (49.2)	1050 (53.0)	1033 (55.93)	1118 (53.52)	3717 (53.66)	717 (51.99)	66.8 (< 0.001)
Country—*n* (%)								416.1 (< 0.001)
England	25,091 (83.6)	12,648 (80.1)	1604 (80.9)	1622 (87.8)	1777 (85.1)	6228 (89.8)	1212 (87.9)	
Scotland	2259 (7.5)	1472 (9.3)	179 (9.0)	102 (5.5)	139 (4.9)	311 (4.5)	56 (4.1)	
Wales	1392 (4.6)	831 (5.3)	97 (4.9)	63 (3.4)	104 (4.9)	231 (3.33%)	66 (4.8)	
Northern Ireland	1283 (4.3)	840 (5.3)	103 (5.2)	60 (3.3)	69 (3.3)	166 (2.39%)	45(3.3)	
Low income—*n* (%)	15,010 (49.9)	6852 (43.4)	710 (35.8)	771 (41.7)	1291 (61.8)	4619 (66.6)	767 (55.6)	1400 (< 0.001)
Ethnicity—*n* (%)								2300 (< 0.001)
White British/Irish	23,435 (78.1)	13,642 (86.4)	1639 (82.7)	1341 (72.6)	1571 (75.2)	4198 (60.5)	1044 (75.7)	
Other White background	882 (2.9)	331 (2.1)	60 (30.3)	55 (2.9)	61 (2.9)	337 (4.9)	38 (2.8)	
Mixed background	502 (1.7)	168 (1.1)	25 (1.3)	50 (2.7)	37 (1.8)	201 (2.9)	21 (1.5)	
Indian	1,044 (3.5)	460 (2.9)	48 (2.4)	64 (3.5)	81 (3.9)	342 (4.9)	49 (3.6)	
Pakistani	782 (2.6)	301 (1.9)	44 (2.2)	52 (2.8)	69 (3.3)	274 (3.9)	42 (3.1)	
Bangladeshi	583 (1.9)	113 (0.7)	20 (1.0)	54 (2.9)	42 (2.0)	318 (4.6)	36 (2.6)	
Black Caribbean	841 (2.8)	322 (2.0)	40 (2.0)	73 (3.9)	82 (2.0)	279 (4.02)	45 (3.3)	
Black African	954 (3.2)	169 (1.1)	47 (2.4)	91 (4.9)	74 (3.5)	518 (7.5)	55 (3.9)	
Other non-White background	1002 (3.3)	285 (1.8)	60 (3.0)	67 (3.6)	72 (3.5)	469 (6.8)	49 (3.6)	

### Missing data

The proportion of missing data at Wave 1 for each variable can be found in the supplemental material (Supplemental material, T1). Almost all the variables were complete at Wave 1, with the exception of sex, which had a very small proportion of missing data. Regarding the primary outcome, 64.8% of participants had missing GHQ data at Wave 10. Participants with missing data on GHQ and sex were more likely to belong to a minority ethnic group, spend 30% or more of their household income on housing costs, be older, have a net household income below the sample median, and be from a country other than England at Wave 1 (Supplemental material, T3). The main exposure, housing affordability problems, was complete at baseline, but had increasing missingness at subsequent waves, with 61.5% missing by Wave 9; participants with partial missing housing affordability data were included in the trajectory analysis due to the use of maximum likelihood estimation. Missing housing affordability data at Wave 9 were predicted by age, country, housing affordability problems at Wave 1, GHQ at Wave 1, low income, and ethnicity; being Black African or from other non-White ethnic backgrounds were the strongest predictors (Supplemental material, T2).

### Derivation of trajectory model

**Fig. 1:**
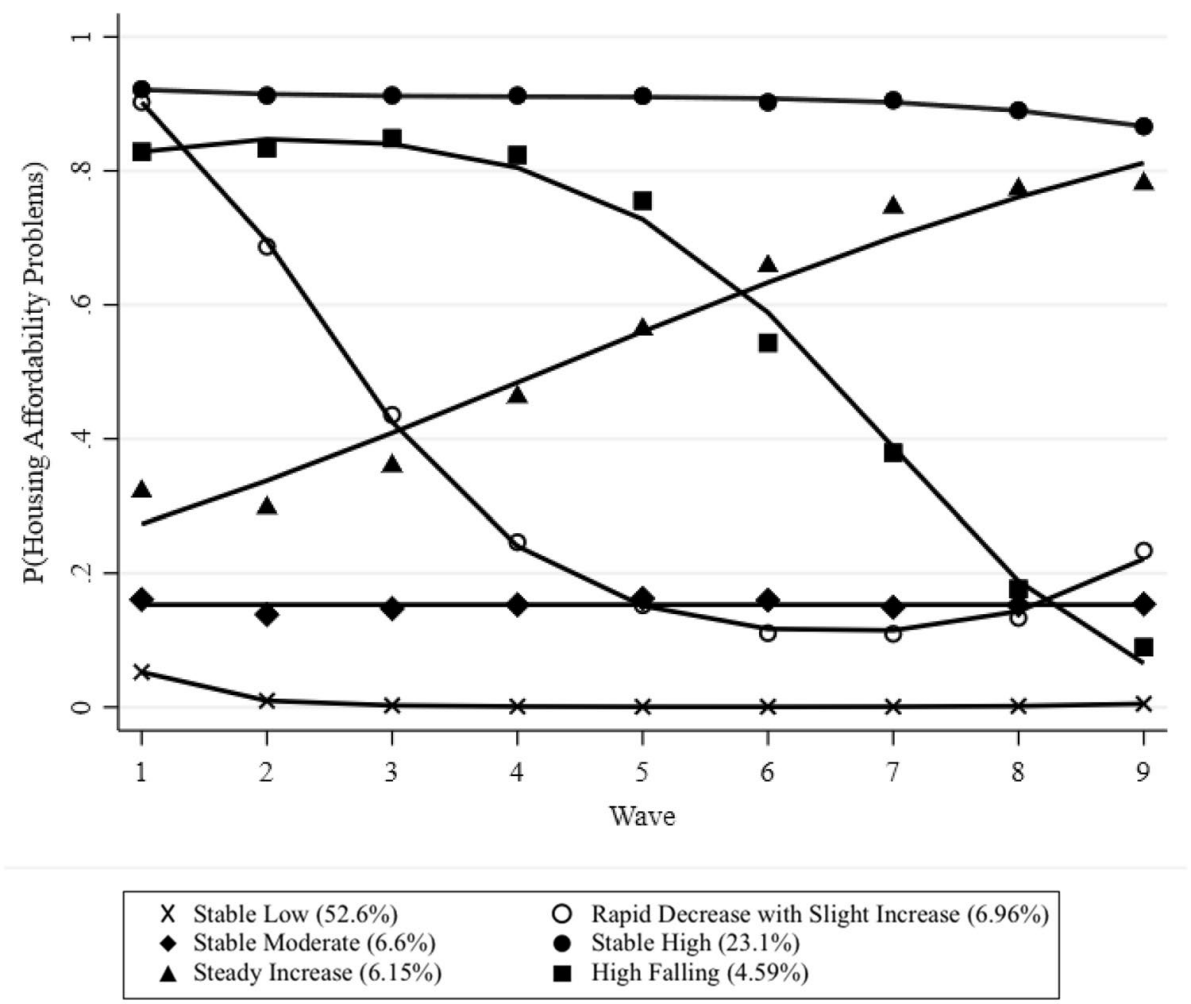
Trajectories of housing affordability problems over nine data waves

The trajectory modelling suggested a six-group solution provided the best fit to the data, with qualitatively distinct patterns of exposure. BIC continued to improve with additional groups; however, the addition of more than 6 groups rendered the model unstable. Comparisons of fit criteria with models of different group numbers, as well as the parameter estimates for the final model, can be found in the supplemental material (Supplemental material, T4–5). The trajectories for the six groups can be seen in Fig. 1. We labelled the groups according to the probability of group members having housing affordability problems over their trajectory. The ‘stable low’ group included more than half the sample, and followed a consistently low probability of facing a housing affordability problem. The ‘stable moderate’ group faced a slightly higher probability of spending 30% or more of their household income on housing costs. The ‘steady increase’ group followed a linear trajectory progressing from a low to high probability of housing affordability problems over the course of nine waves. The ‘rapid decrease with slight increase’ group quickly fell to a lower probability of housing affordability problems, showing the beginnings of an upturn in Wave 8 (2016–2018). The ‘stable high’ group was the second largest, characterised by a consistently elevated probability of housing affordability problems. Finally, the ‘high falling’ group was exposed to approximately 4 years of higher affordability problems, then decreasing to a moderate level after Wave 4 (2012–2014).

The results of the sensitivity analysis using participants with complete housing affordability data can be found in the supplemental material (Supplemental material, F2). The sensitivity analysis also found a six-group solution to be the best fit to the data, with similar trajectories. However, the ‘rapid decrease with slight increase’ trajectory did not show a slight increase. Furthermore, the ‘high falling’ trajectory did not exhibit a higher probability of housing affordability problems for as long, with a more rapid decrease to a moderate probability. The ‘stable high’ and ‘high falling’ groups were much smaller.

The distribution of the sample characteristics within the six trajectory groups can be found in Table 1. Each group presented an approximately equal split by sex, with a slightly higher proportion of females in the ‘steady increase’ group. The ‘stable low’ group had the oldest participants and the highest proportion of White British/Irish participants. The youngest group was the ‘stable high’ group, the group with the highest proportion of English participants and the most participants with a household income below the sample median. The ‘stable low’ group also had the lowest proportion of White British/Irish participants and the highest proportion of Indian, Pakistani, Bangladeshi, Black Caribbean, and Black African participants, as well as participants from mixed backgrounds, other White backgrounds, and other non-White backgrounds. At Wave 1, the ‘rapid decrease with slight increase’ group had a mean GHQ score of 12.1, the highest mean GHQ score. The ‘high falling’ group had the highest mean GHQ at Wave 10, with an average score of 12.4, approximately two points higher than the group with the lowest score, the ‘stable low’ group. Comparison of the sample characteristics showed that the differences between trajectory groups were statistically significant.

### Housing affordability problems trajectories and mental health scores at follow-up

Table 2 shows the results of the linear regression model estimating the association between the housing affordability problems trajectory groups and the primary outcome of Wave 10 GHQ total score. Compared to those with 'stable low’ housing affordability problems, all other trajectories were associated with significantly worse mental health at follow-up. People facing successive time points with high probability of affordability problems were more likely to experience worse mental health, especially the ‘stable high’ group and the ‘high falling’ group. The smallest association was seen in the ‘stable moderate’ group.

**Table 2:** Linear regression model results for the association between trajectories of housing cost burden and GHQ

Model	Unadjusted model		Partially adjusted^a^		Fully adjusted^b^	
	Coeff. (95% CI)	*p* value	Coeff. (95% CI)	*p *value	Coeff. (95% CI)	*p *value
Stable low	Reference					
Stable moderate	0.91 (0.57, 1.24)	< 0.001	0.58 (0.24, 0.92)	0.001	0.59 (0.26, 0.94)	0.001
Steady increase	1.027 (9.68, 1.38)	< 0.001	0.56 (0.22, 0.9)	0.001	0.49 (0.15, 0.83)	0.005
Rapid decrease with slight increase	1.02 (0.63, 1.41)	< 0.001	0.63 (0.25, 1.01)	0.001	0.35 (-0.026, 0.72)	0.068
Stable high	1.43 (1.14, 1.73)	0.15	0.93 (0.63, 1.22)	< 0.001	0.56 (0.26, 0.86)	< 0.001
High falling	1.69 (1.16, 2.22)	< 0.001	1.29 (0.76, 1.82)	< 0.001	1.06 (0.53, 1.58)	< 0.001

^a^Adjusted for age, sex, and country

^b^Adjusted for age, sex, country, and low income

After adjusting for age, sex, and country at Wave 1, evidence for an association persisted for all groups, however with smaller effect sizes. For example, compared to the ‘stable low’ group, the ‘stable high’ group had a GHQ score on average 0.93 points higher (95% CI 0.63–1.22), whilst the ‘high falling’ group had a GHQ score on average 1.29 points higher (95% CI 0.76–1.28) These results were further attenuated for all trajectory groups after adjusting for median household income. However, evidence of an association with GHQ remained for all but the ‘rapid decrease with slight increase’ group. The ‘high falling’ group continued to have the largest effect size, with a GHQ score on average 1.06 (95% 0.53–1.58) points higher.

The sensitivity analysis comparing the linear regression results between a complete case analysis and the imputed analysis showed the same pattern of results (Supplemental material, T6). Similarly, including ethnicity in the linear regression model did not attenuate the results (Supplemental material, T7). The sensitivity analysis adjusting for Wave 1 GHQ showed the association between housing cost burden and mental health was partially explained by baseline mental health; however, the results remained significant for all trajectory groups (Supplemental material, T8). The effect sizes were further attenuated after full adjustment, but the ‘high falling’ and ‘stable moderate’ groups remained significant.

## Discussion

In a large population-based cohort, we found that trajectories of housing affordability problems over nine years were associated with worse mental health. The mental health impact was largest for groups exposed to current or past cumulative affordability problems. This association persisted after controlling for age, sex, country, and median household income. To our knowledge, this is the first study to examine trajectories of housing affordability problems and mental health.

These results demonstrate that exposure to cumulative years of housing affordability problems was associated with worse mental health than persistent exposure to few or no affordability problems. Whilst the levels of mental health appear to be similar across trajectory groups, small changes in GHQ become meaningful at the population level; after adjustment, the ‘high falling’ group had on average over a one point greater GHQ score than the ‘stable low’ group. The worse mental health associated with housing affordability problems may be partly explained by deprivation due to a lack of resources available to meet other needs related to wellbeing [13, 24]. Furthermore, with a high burden, individuals may be forced to make trade-offs, for example, on neighbourhood or housing quality [24], which in turn further exacerbates mental health problems [8, 25]. The limited longitudinal research on various measures of housing affordability and mental health is consistent with these findings; other studies have found a dose–response effect of increasing cumulative years of affordability problems having an increasingly negative mental health impact [11, 15, 26, 27]. Our study advances these findings by applying group-based trajectory modelling, which allows us to identify existing latent sub-groups following different trajectories in the study population, rather than fitting the population into predetermined groups of different dose levels.

We found a stronger association between housing cost burden and worse mental health for the ‘high falling’ group, compared to the ‘stable low’ group. The ‘high falling’ group did not have the worst mental health at Wave 1, nor did this group present greater risk factors for worse mental health (including ethnicity or low-income status), compared to the other trajectory groups. Therefore, the results suggest that the worse mental health found in the ‘high falling’ group may be better explained by their distinct experience of housing affordability over the ten-year study period, rather than the group composition. The literature on socioeconomic insecurity suggests that whilst individuals can heal from past insecurities, the accumulation of these past insecurities can have an increasing negative impact in the present, regardless of insecurity in the present [28]. In the case of unemployment, for example, past unemployment spells may lead to constant fear of returning to this state [29]. The results of the present study suggest that the ‘high falling’ group has not ‘healed’ from a past period of housing affordability problems and continues to have a sustained mental health impact, even in the absence of current problems. This finding underscores the importance of taking into account past housing affordability problems when targeting mental health support interventions; considering present housing affordability status alone obscures the long-term mental health impact of a history of insecurity.

Whilst the sensitivity analysis adjusting for baseline mental health showed an attenuation of the effect sizes for all trajectory groups, Wave 1 GHQ does not appropriately capture pre-existing mental health as it is measured 10 years before the outcome and does not account for the time-varying nature of mental health. Furthermore, evidence of an association between mental health and housing affordability problems remained for the ‘high falling’ group, even after full adjustment.

After adjusting for household income, evidence of a negative mental health impact remained for all but one group. This finding is aligned with the literature, as studies that have assessed the impact of various sources of personal debt have similarly found an impact on mental health for housing debt, independent of other financial problems [30–32]. A relatively large difference in GHQ score was also seen in the ‘stable high’ group; however, this effect was attenuated more than any other group after adjusting for median household income. As this trajectory group has the biggest proportion of low-income participants, it may be that these participants do not experience a mental health impact of a housing affordability problems over and above the mental health impact of financial stress due to their income status. The ‘rapid decrease with slight increase’ group was also greatly attenuated. Whilst the finding for these two groups is at odds with the aforementioned literature, further research is needed to understand whether there is a particular threshold of general economic insecurity that, once surpassed, does not differentiate between sources of insecurity.

The distribution of ethnicity and age differed across trajectory groups. The high number of participants from Indian, Pakistani, Bangladeshi, Black Caribbean, Black African, and mixed ethnic backgrounds, as well as other White backgrounds and other non-White backgrounds following a ‘stable high’ trajectory of a housing affordability problems is consistent with the UK census data: Black, Asian and minority ethnic groups are more likely to face housing deprivation, in particular Gypsy or Irish travellers, Black Africans, and Bangladeshis [33]. The sensitivity analysis adjusting for ethnicity suggested that ethnicity did not explain the relationship between housing affordability and mental health, but future research should explore potential effect modification. The same ‘stable high’ trajectory group also had the youngest mean age. These results reflect the reduction in access to stable and affordable housing for ‘generation rent’ [34]. According to the Office for National Statistics, there is an increasing number of people in their mid-30 s to mid-40 s in the private rental sector, the most expensive sector in terms of housing costs, compared to older adults [35]. Future research should consider the potential differential mental health effects of housing affordability for these sub-groups.

This study has several strengths and limitations. It benefits from a large longitudinal cohort that is representative of the UK population. As this cohort has been followed since 2009, this study contributes to the understanding of the impact of the 2007–2009 global financial crisis. Additionally, to our knowledge, this study represents the first application of a group-based trajectory analysis on housing affordability. This application is a strength as it has the benefit of capturing the dynamic changes in housing affordability over time for distinct groups, and therefore allows us to assess how these unique patterns impact mental health.

Attrition in this study meant that only approximately 40% of participants provided housing affordability data at all nine waves included in the trajectory analysis. The results of the sensitivity analysis indicate however that a similar trajectory modelling solution was appropriate for both the complete case sample and the imputed total analysis sample. The small differences in the shapes of the ‘high falling’ and ‘rapid decrease with slight increase’ trajectories may be explained by the omission of more than half of the analysis sample. The sensitivity analysis does not include many participants exposed to more affordability problems; participants that were non-respondents by Wave 9 were more likely to have been categorised as facing housing affordability problems at Wave 1. The maximum likelihood estimation used in the trajectory modelling allows us to use these participants in the full analysis and take into account their partial information to derive more appropriate trajectories. Attrition also led to a large proportion of missing mental health data at follow-up; approximately two-thirds of the cohort were without complete outcome data. However, multiple imputation has been shown to reduce bias, even with high proportions of missing data; this bias is reduced especially with the inclusion of complete auxiliary variables, which were used in this study [36].

Whilst housing affordability problems are measured over nine waves of data, we took covariates from only one wave, potentially obscuring their time-varying nature. However, we considered sex and country to be approximately stable, with minimal inconsistencies across the data waves. Age increased at the same rate for all participants, so that the differences in age would be equal at all waves. Although household income varied, the inclusion of the wave one measure provided an approximation of household income status at the start of the trajectories.

Finally, there is some variability and debate in measurements of housing affordability, with criticisms of the 30% cut-off used in this study that suggest it does not account for how this percentage may represent a different financial burden for households of different compositions or incomes [37]. However, this study is specifically interested in the impact of housing affordability problems on mental health, and by taking into account other financial difficulties in this measure, it becomes difficult to isolate the housing effects. Furthermore, this study is of interest to stakeholders in public health and housing; as a cut-off of 30% is the most widely used measure of housing affordability, it was applied to allow for clear interpretation and comparison in the context of other research, and for policy-relevant conclusions to be made about the impact of affordability problems. Future research could investigate the mental health impact on different types of households in more detail.

## Conclusion

This study provides evidence of a long-term negative mental health impact of exposure to housing affordability problems, even after a more recent decrease in affordability problems. The results support the need for mental health interventions that not only target populations currently experiencing housing affordability problems, but also those that may have been exposed to accumulation of a burden in the past and are still experiencing psychological distress in the present.

## Supplementary Information

Below is the link to the electronic supplementary material.

Supplementary file1 (DOCX 142 KB)
