# Supplementary material for: Trajectories of housing affordability and mental health problems: a population-based cohort study
Source: Soc Psychiatry Psychiatr Epidemiol. 2022 Jun 29;58(5):769–78. doi: 10.1007/s00127-022-02314-x (PMC10097755; doi:10.1007/s00127-022-02314-x)
Supplement: Supplementary file 1 — Supplementary file1 (DOCX 142 KB) [file 127_2022_2314_MOESM1_ESM.docx]

**Supplemental Material**

Table 1. Missing data on sample characteristics

| Variable | Missing — n (%) |
| --- | --- |
| Age | 0 |
| Female | 26 (0.09) |
| Country | 0 |
| Housing affordability problems (Wave 1) | 0 |
| GHQ (Wave 10) | 19,448 (64.9) |
| Below median household income | 0 |
| Ethnicity | 0 |

Table 2. Predictors of missing housing affordability data at Wave 9

| Variable | Odds Ratio (95% CI) | p-value | SE |
| --- | --- | --- | --- |
| Age | 0.0019 (0.00014, 0.0036) | 0.035 | 0.088 |
| Country |  |  |  |
| England | Reference |  |  |
| Scotland | 0.22 (0.12, 0.31) | <0.001 | 0.049 |
| Wales | 0.24 (0.11, 0.36) | <0.001 | 0.063 |
| Northern Ireland | 0.087 (-0.0399, 0.21) | 0.18 | 0.065 |
| GHQ at Wave 1 | 0.011 (0.0067, 0.016) | <0.001 | 0.0024 |
| Housing Affordability Problems at Wave 1 | 0.18 (0.12, 0.24) | <0.001 | 0.032 |
| Low Income | 0.35 (0.3, 0.41) | <0.001 | 0.028 |
| Ethnicity |  |  |  |
| White British/Irish | Reference |  |  |
| Other White background | 0.42 (0.25, 0.58) | <0.001 | 0.084 |
| Mixed background | 0.34 (0.13 0.55) | 0.001 | 0.11 |
| Indian | 0.41 (0.25, 0.56) | <0.001 | 0.0799 |
| Pakistani | 0.32 (0.13, 0.52) | 0.001 | 0.1 |
| Bangladeshi | 0.48 (0.23, 0.74) | <0.001 | 0.13 |
| Black Caribbean | 0.45 (0.27, 0.63) | <0.001 | 0.091 |
| Black African | 0.9 (0.71, 1.09) | <0.001 | 0.099 |
| Other non-White background | 0.72 (0.55, 0.89) | <0.001 | 0.088 |

Figure 1. Sample inclusions and exclusions


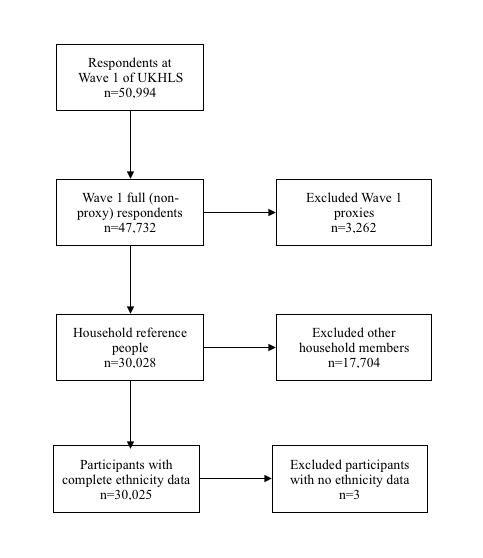


Table 3. Sample characteristics of complete cases compared to cases with missing GHQ and sex

| Variable | Complete cases  (n=10577) | Missing GHQ and sex  (n=19488) |
| --- | --- | --- |
| GHQ (Wave 10) — mean (SD) | 11.2 (5.5) | - |
| Housing cost burden (Wave 1) — mean (SD) | 3286 (31.1) | 7432 (38.2) |
| Age — mean (SD) | 49.7 (14.2) | 50.2 (18.4) |
| Female — n (%) | 5420 (51.2) | 9973 (51.4) |
| Country — n (%) |  |  |
| England | 8951 (84.6) | 16140 (82.9) |
| Scotland | 781 (7.4) | 1,478 (7.6) |
| Wales | 410 (3.9) | 982 (5.1) |
| Northern Ireland | 435 (4.1) | 848 (4.4) |
| Low Income — n (%) | 4375 (41.4) | 10635 (54.7) |
| Ethnicity — n (%) |  |  |
| White British/Irish | 9028 (85.4) | 14407 (74.1) |
| Other White background | 235 (2.2) | 647 (3.3) |
| Mixed background | 156 (1.5) | 346 (1.8) |
| Indian | 277 (2.6) | 767 (3.9) |
| Pakistani | 198 (1.9) | 584 (3) |
| Bangladeshi | 107 (1.0) | 476 (2.5) |
| Black Caribbean | 206 (1.9) | 635 (3.3) |
| Black African | 171 (1.6) | 783 (4.0) |
| Other non-White background | 199 (1.9) | 803 (4.1) |

Table 4. Comparison of fit statistics for trajectory models with different cluster numbers

| Number of Groups | Bayesian Information Criterion |
| --- | --- |
| 1 | -101448.96 |
| 2 | -74418.16 |
| 3 | -72263.05 |
| 4 | -71152.2 |
| 5 | -71051.4 |
| 6 | -70735.3 |

Table 5. Parameter estimates and standard errors for trajectories in main analysis

| n=30,025 | Stable Low | Stable Moderate | Steady Increase | Rapid Decrease with Slight Increase | Stable High | High Falling |
| --- | --- | --- | --- | --- | --- | --- |
| Intercept | -0.44 (0.49) | -1.8 (0.12) | -1.28 (0.19) | 3.82 (0.295) | 2.63 (0.24) | 1.21 (0.42) |
| Linear | -2.69 (0.54) | - | 0.302 (0.032) | -1.79 (0.15) | -0.21 (0.2) | 0.44 (0.28) |
| Quadratic | 0.23 (0.054) | - | - | 0.14 (0.013) | 0.048 (0.048) | -0.097 (0.034) |
| Cubic | - | - | - | - | -0.0038 (0.0032) | - |

Figure 2. Trajectories of housing affordability problems over nine data waves for participants with complete housing affordability data (n=12,063)


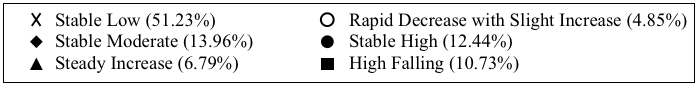


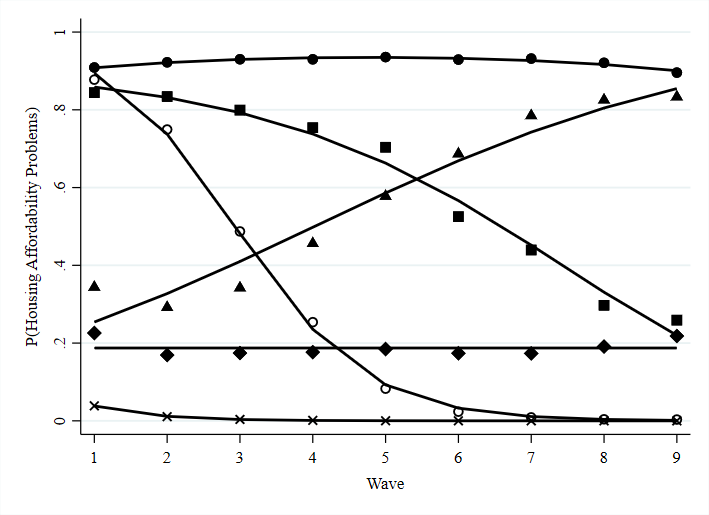


Table 6. Linear regression model results for the association between trajectories of housing cost burden and GHQ: comparison of complete cases and multiple imputation

| Model | Complete Case Analysis | | | Multiple Imputation | | |
| --- | --- | --- | --- | --- | --- | --- |
|  | Coeff. (95% CI) | p-value | SE | Coeff. (95% CI) | p-value | SE |
| **Unadjusted** |  |  |  |  |  |  |
| Stable Low | Reference |  |  | Reference |  |  |
| Stable Moderate | 1.22 (0.85, 1.59) | <0.001 | 0.19 | 0.91 (0.57, 1.24) | <0.001 | 0.17 |
| Steady Increase | 1.31 (0.87, 1.74) | <0.001 | 0.22 | 1.027 (9.68, 1.38) | <0.001 | 0.18 |
| Rapid Decrease with Slight Increase | 0.91 (0.46, 1.36) | <0.001 | 0.23 | 1.02 (0.63, 1.41) | <0.001 | 0.19 |
| Stable High | 1.84 (1.45, 2.23) | <0.001 | 0.201 | 1.43 (1.14, 1.73) | 0.15 | 1.16 |
| High Falling | 2.21 (1.55, 2.87) | <0.001 | 0.34 | 1.69 (1.16, 2.22) | <0.001 | 0.27 |
| **Partially adjusted^1^** |  |  |  |  |  |  |
| Stable Low | Reference |  |  |  |  |  |
| Stable Moderate | 0.798 (0.41, 1.19) | <0.001 | 0.198 | 0.58 (0.24, 0.92) | 0.001 | 0.17 |
| Steady Increase | 0.71 (0.26, 1.16) | 0.002 | 0.23 | 0.56 (0.22, 0.9) | 0.001 | 0.17 |
| Rapid Decrease with Slight Increase | 0.44 (-0.018, 0.9) | 0.06 | 1.88 | 0.63 (0.25, 1.01) | 0.001 | 0.19 |
| Stable High | 1.24 (0.83, 1.65) | <0.001 | 0.21 | 0.93 (0.63, 1.22) | <0.001 | 0.15 |
| High Falling | 1.72 (1.08, 2.35) | <0.001 | 0.32 | 1.29 (0.76, 1.82) | <0.001 | 0.27 |
| **Fully adjusted^2^** |  |  |  |  |  |  |
| Stable Low | Reference |  |  |  |  |  |
| Stable Moderate | 0.79 (0.39, 1.17) | <0.001 | 0.198 | 0.59 (0.26, 0.94) | 0.001 | 0.17 |
| Steady Increase | 0.62 (0.17, 1.078) | 0.007 | 0.23 | 0.49 (0.15, 0.83) | 0.005 | 0.17 |
| Rapid Decrease with Slight Increase | 0.204 (-0.26, 0.66) | 0.38 | 0.23 | 0.35 (-0.026, 0.72) | 0.068 | 0.19 |
| Stable High | 0.94 (0.52, 1.36) | <0.001 | 0.21 | 0.56 (0.26, 0.86) | <0.001 | 0.15 |
| High Falling | 1.48 (0.87, 2.099) | <0.001 | 0.31 | 1.06 (0.53, 1.58) | <0.001 | 0.27 |

^1^ Adjusted for age, sex, and country; ^2^ Adjusted for age, sex, country, and low income

Table 7. Linear regression model results for the association between trajectories of housing cost burden and GHQ: further adjustment for ethnicity

| Trajectory Group | Unadjusted | | Partially Adjusted^1^ | | Full Adjusted^2^ | |
| --- | --- | --- | --- | --- | --- | --- |
|  | Coeff. (95% CI) | p-value | Coeff. (95% CI) | p-value | Coeff. (95% CI) | p-value |
| Stable Low | Reference |  |  |  |  |  |
| Stable Moderate | 0.91 (0.57, 1.24) | <0.001 | 0.85 (0.51, 1.19) | <0.001 | 0.56 (0.23, 0.89) | 0.001 |
| Steady Increase | 1.027 (9.68, 1.38) | <0.001 | 1.04 (0.68, 1.41) | <0.001 | 0.52 (0.13, 0.92) | 0.01 |
| Rapid Decrease with Sight Increase | 1.02 (0.63, 1.41) | <0.001 | 0.98 (0.6, 1.36) | <0.001 | 0.33 (-0.041, 0.69) | 0.081 |
| Stable High | 1.43 (1.14, 1.73) | 0.15 | 1.43 (1.11, 1.75) | <0.001 | 0.59 (0.28, 0.91) | <0.001 |
| High Falling | 1.69 (1.16, 2.22) | <0.001 | 1.64 (1.17, 2.12) | <0.001 | 1.03 (0.57, 1.49) | <0.001 |

^1^Adjusted for ethnicity ^2^Adjusted for ethnicity, age, sex, country, and low income

Table 8. Linear regression model results for the association between trajectories of housing cost burden and GHQ: further adjustment for Wave 1 GHQ

| Trajectory Group | Unadjusted | | Partially Adjusted^1^ | | Full Adjusted^2^ | |
| --- | --- | --- | --- | --- | --- | --- |
|  | Coeff. (95% CI) | p-value | Coeff. (95% CI) | p-value | Coeff. (95% CI) | p-value |
| Stable Low | Reference |  |  |  |  |  |
| Stable Moderate | 0.91 (0.57, 1.24) | <0.001 | 0.68 (0.35, 1.006) | <0.001 | 0.37 (0.054, 0.69) | 0.022 |
| Steady Increase | 1.027 (9.68, 1.38) | <0.001 | 0.76 (0.41, 1.12) | <0.001 | 0.27 (-0.12, 0.65) | 0.17 |
| Rapid Decrease with Sight Increase | 1.02 (0.63, 1.41) | <0.001 | 0.44 (0.092, 0.79) | 0.013 | -0.098 (-0.45, 0.25) | 0.58 |
| Stable High | 1.43 (1.14, 1.73) | 0.15 | 0.98 (0.69, 1.27) | <0.001 | 0.27 (-0.022, 0.56) | 0.07 |
| High Falling | 1.69 (1.16, 2.22) | <0.001 | 1.14 (0.69, 1.6) | <0.001 | 0.62 (0.17, 1.063) | 0.007 |

^1^Adjusted for Wave 1 GHQ ^2^Adjusted for Wave 1 GHQ, age, sex, country, low income
